# Jaeumganghwa-Tang, a traditional herbal formula, improves muscle function and attenuates muscle loss in aged mice

**DOI:** 10.20463/jenb.2017.0059

**Published:** 2017-03-31

**Authors:** Yun Mi Lee, Ohn Soon Kim

**Affiliations:** 1 KM Convergence Research Division, Korea Institute of Oriental Medicine, Daejeon Republic of Korea

**Keywords:** Sarcopenia, Jaeumganghwa-Tang, Muscle loss, TGF-β

## Abstract

**[Purpose]:**

Jaeumganghwa-Tang (JGT), a traditional herbal formula composed of 12 medicinal herbs, is used for the treatment of age-related diseases. In the present study, we investigated the effects of JGT on muscle mass and function in aged mice.

**[Methods]:**

Young (5-month-old) and old (19-month-old) male C57BL/6 mice were divided into two groups each; one group received JGT (75 mg/d) and the other group received the vehicle for 6 weeks. At the end of the experimental period, muscle strength was examined using the wire hang test, and the tibialis anterior and gastrocnemius muscles were weighed. Muscle samples were further used for histological analysis to assess muscle damage, and the expression of transforming growth factor-beta was investigated via western blotting and immunohistochemistry.

**[Results]:**

Our results showed that treatment of old mice with JGT improved muscle strength, increased skeletal muscle mass, alleviated muscle damage, and suppressed intramuscular expression of transforming growth factor-beta.

**[Conclusion]:**

In conclusion, JGT has beneficial effects on age-related loss of muscle mass and function. Thus, it might serve as a potential therapeutic agent for sarcopenia.

## INTRODUCTION

Sarcopenia is an age-related muscle disorder characterized by the loss of skeletal muscle mass and function^1, 2^. Sarcopenia comprises myopenia (a decline in muscle mass) and dynapenia (a decline in muscle strength); thus, it leads to compromised physical function, high risk of disability, and low quality of life^3^. According to the literature, the prevalence of sarcopenia in people aged 60–70 years old is 5–13%, while it ranges from 11 to 50% in people older than 80 years^4^. Current estimates suggest that approximately 200 million people worldwide suffer from sarcopenia to a degree that affects their life’s quality^5^. Thus, the development of therapeutic strategies for the management of sarcopenia is highly required. 

Jaeumganghwa-tang (JGT, *Zi-ying-jiang-huo-tang* in Chinese and *Jin-koka-to* in Japanese) is one of the most widely prescribed traditional herbs in East Asia. JGT is composed of 12 medicinal herbs: Paeoniae Radix, Angelicae Gigantis Radix, Rehmanniae Radix Preparata, Atractylodis Rhizoma Alba, Liriopis Tuber, Rehmanniae Radix Crudus, Citri Unshius Pericarpium, Anemarrhenae Rhizoma, Phellodendri Cortex, Glycyrrhizae Radix et Rhizoma, Zingiberis Rhizoma Crudus, and Zizyphi Fructus^6, 7^. It has been mentioned in the Donguibogam, a traditional Korean medical book compiled by Heo Jun (1539–1615), that JGT can be prescribed for the treatment of “kidney deficiency,” which is a concept in traditional Korean medicine that refers to age-related pathophysiology such as lack of energy, vision, hearing, and muscle function. Recent studies have investigated the pharmacological effects of JGT against allergic inflammatory reactions and mechanisms of cancer^8, 9^. In addition, JGT was found to inhibit the development of benign prostatic hyperplasia (BPH) in a rat model^10^. Despite the potential therapeutic benefit of JGT as an anti-aging drug, only few studies have investigated its effect on sarcopenia. 

Thus, in this study, we aimed to investigate the effects of JGT on muscle mass and function in aged mice. To elucidate the underlying molecular mechanisms, we assessed the protein expression of TGF-β in the muscle tissue. 

## METHODS

### Animal treatment

Young and old male C57BL/6 mice (aged 5 and 19months, respectively) were obtained from the LaboratoryAnimal Resource Center in Korea Basic Science Institute(KBSI, Gwangju, Korea) and housed in a specific pathogen-free facility under constant conditions (12 h lightdarkcycle at 22 ± 1 °C and 55 ± 5% humidity). After anacclimation period of 1 week, the mice were randomlydivided into four groups as follows: young mice administeredvehicle (Y:Con, n = 4); young mice administeredJGT (Y:JGT, n = 4); old mice administered vehicle(O:Con, n = 8); and old mice administered JGT (O:JGT, n= 8). JGT was purchased from Han Kook Shin Yak PharmaceuticalCo., Ltd. (Chungnam, Korea) in the form ofpowdered granules. The chemical composition of thesegranules was verified based on the company standards(Table 1). JGT was dissolved in saline and orally administeredto the mice at a dose of 75 mg/d for 6 weeks. Thisdose was calculated from the adult human dose (15 g/d)^11^. At the end of the experimental period, muscle strengthwas examined; then, mice were anesthetized with diethylether and killed, and muscle tissues were collected. Allanimal experiments were performed in accordance withthe guidelines of the Animal Care and Use Committee ofthe Korea Institute of Oriental Medicine (KIOM, Daejeon,Korea) with reference number #15-083. 

**Table 1:** The components of JGT and their compounded rates.

Scientific name	Latin name	Compounded rates (%)
*Angelica gigas*	Angelicae Gigantis Radix	10.2
*Paeonia lactiflora*	Paeoniae Radix	10.2
*Rehmannia glutinosa*	Rehmanniae Radix Crudus	10.2
*Asparagus cochinchinensis*	Asparagi Tuber	10.2
*Atractylodes japonica*	Atractylodis Rhizoma Alba	12.3
*Anemarrhena asphodeloides*	Anemarrhenae Rhizoma	6.1
*Phellodendron amurense*	Phellodendri Cortex	6.1
*Glycyrrhiza uralensis*	Glycyrrhizae Radix et Rhizoma	6.1
*Zizyphus jujube*	Zizyphi Fructus	4.1
*Zingiber officinale*	Zingiberis Rhizoma Crudus	4.1
*Liriope platyphylla*	Liriopis Tuber	10.2
*Citrus unshiu*	Citri Unshius	10.2

### Wire hang test

A wire mesh grid (15 × 25 cm) was used to measure the muscle strength as follows; the mouse was placed on the wire mesh grid 40 cm above a foam cushion, then the mesh was inverted so that the mouse is forced to hang to the wire using its four limbs. The timer was activated upon inversion and the hanging time was recorded as the duration for which the mouse keeps hanging before falling on the cushion^12^. 

### Tissue collection

The tibialis anterior (TA) and gastrocnemius (GC) muscles were immediately dissected and washed in phosphate-buffered saline (PBS) after anesthesia. Then, excess PBS was removed by blotting on a paper towel and the muscles were weighed using an analytical scale (Mettler-Toledo International Inc., model# ML204T). For protein extraction, tissues were snap-frozen in liquid nitrogen and stored at −80 °C; for histology (hematoxylin and eosin (H&E) staining) and immunohistochemistry, tissues were fixed via overnight immersion in 10% phosphate-buffered formalin. 

### Western blot analysis of muscle tissues

Western blot assays were carried out using total cellular protein. The frozen muscle was homogenized in RIPA buffer (20 mL/g of tissue) (Thermo Scientific, Rockford, IL) containing protease and phosphatase inhibitors according to the manufacturer’s instructions. The protein concentration was determined using a Bio-Rad Protein Assay reagent (Bio-Rad, Hercules, CA). Equal amounts of protein extracts (20 μg) were resolved via 4–20% sodium dodecyl sulfate-polyacrylamide gel electrophoresis (SDS-PAGE) and transferred to a polyvinylidene fluoride (PVDF) membrane. The membrane was incubated with a blocking solution (5% skimmed milk in Tris-buffered saline containing Tween 20 (TBST)) and incubated overnight at 4 °C with the appropriate primary antibody; TGF-β (Abcam, Boston, MA) and glyceraldehyde 3-phosphate dehydrogenase (GAPDH) (Santa Cruz Biotechnology, Dallas, TX). The membranes were washed three times with TBST and incubated with a horseradish peroxidase (HRP)-conjugated secondary antibody (Jackson ImmunoResearch, West Grove, PA) for 1 h at room temperature. Then, they were washed again with TBST for three times and developed signals were detected using an enhanced chemiluminescence (ECL) kit (Thermo Scientific). Images were captured using Chemi-Doc imaging system (Bio-Rad). 

### Histological analysis of muscle tissues 

Mouse TA and GC muscle tissues were fixed in 10% phosphate-buffered formalin for 24 h and embedded in paraffin; then, 4-μm-thick sections were cut and stained with H&E. To analyze the muscle fiber phenotype, muscle tissues were imaged using a microscope (BX43, Olympus, Japan). 

### Immunohistochemical analysis of muscle tissues

Formalin-fixed paraffin-embedded muscle tissue sections were deparaffinized and rehydrated then boiled in 10 mM sodium citrate, pH 6.0, with 0.05% Tween-20 for 30 min for antigen retrieval. Sections were blocked in normal serum and incubated with TGF-β (1/500) antibodies overnight. Endogenous HRP activity was quenched by incubation with 3% hydrogen peroxide for 10 min. Secondary antibody staining was performed using the Polink-2 Plus HRP kit (GBI Labs, Bothell, WA) and detected with 3,3′-diaminobenzidine (DAB). Negative controls for immunohistochemistry were run by incubating the sections with non-immune serum instead of the primary antibody. 

### Statistical analysis

Data are expressed as the mean ± standard error of the mean (SEM). Data were analyzed using one-way analysis of variance and Dunnett’s multiple comparisons tests using Prism software (GraphPad Software Inc., La Jolla, CA). P values lower than 0.05 indicated statistical significance. 

## RESULTS

### JGT improves muscle strength in aged mice

**Figure 1.**
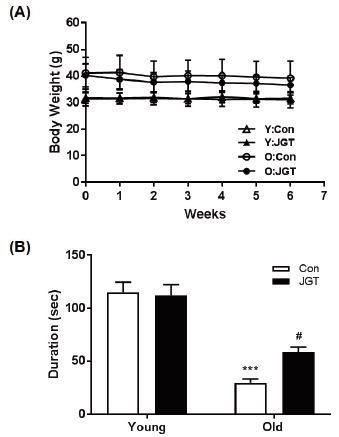
Effects of JGT on body weight and muscle strength in young and old mice. (A) Body weight changes in each group during the experimental period. (B) Muscle strength as determined using the wire hang test in young and old mice. Bar graphs represent the mean ± SEM. ***P < 0.001 vs. young controls, #*P* < 0.05 vs. old controls. JGT, Jaeumganghwa-tang.

In order to examine the effects of JGT treatment on age-associated muscle weakness, both young (5-monthold) and old (19-month-old) mice treated with saline or JGT for 6 weeks were exposed to the wire hang test. During the treatment period, no significant differences in body weights were observed between the young and old control and JGT-treated mice (Fig. 1A). The wire hang test was performed during the last week of the treatment period. We measured the maximum duration for which the mice remained hanging to the inverted wire mesh grid before falling. This duration was significantly shorter in the old mice than in the young mice. However, JGT-treated old mice hung for a longer duration than the old control mice. JGT treatment had no effect on the hanging duration in the young mice groups (Fig. 1B). These results suggest that JGT has beneficial effects on muscle strength. 

### JGT increases muscle mass in aged mice

**Figure 2.**
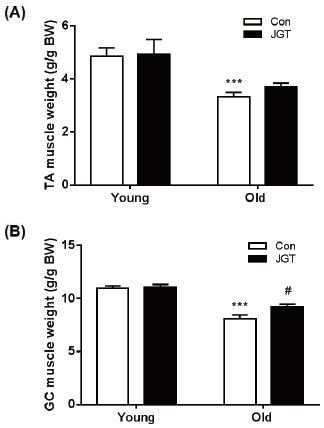
Effects of JGT on muscle weight in young and old mice. The tibialis anterior (A) and gastrocnemius (B) muscles from each experimental group were weighed and normalized to their body weight. Bar graphs represent the mean ± SEM. ***P < 0.001 vs. young controls, #P < 0.05 vs. old controls. JGT, Jaeumganghwa-tang; TA, tibialis anterior; GC, gastrocnemius; BW, body weight.

In order to examine the effect of JGT treatment on age-associated muscle loss, we weighed the TA and GC muscles of all the mice after biopsy. As expected, aging resulted in a significant decline in the weights of both muscles. The JGT-treated old mice demonstrated an increase in the weights of the TA muscles, but this increase was not significant (Fig. 2A). However, JGT treatment significantly increased the weights of the GC muscles in the JGT-treated old mice compared to the old control mice (Fig. 2B). JGT treatment had no effect on the weights of either muscle in the young mice groups. 

### JGT ameliorates muscle damage in aged mice

**Figure 3.**
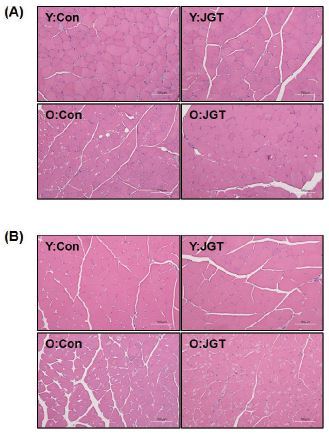
H&E staining images of tibialis anterior (A) and gastrocnemius (B) muscles from each experimental group. Original magnification, ×200. Scale bar, 200 μm. Y:Con, young mice administered vehicle; Y:JGT, young mice administered JGT; O:Con, old mice administered vehicle; O:JGT, old mice administered JGT; JGT, Jaeumganghwa-tang.

Histological analysis of TA and GC muscles via H&E staining revealed significant muscle damage in the old mice groups. As shown in Fig. 3, the muscles of the old mice showed a reduction in the muscle fiber size and an increase in the amount of connective tissue. These age-related muscle changes were found to be significantly improved in the JGT-treated mice compared with the agematched control mice (Fig. 3). 

### JGT reduces expression of TGF-β in aged mice

**Figure 4.**
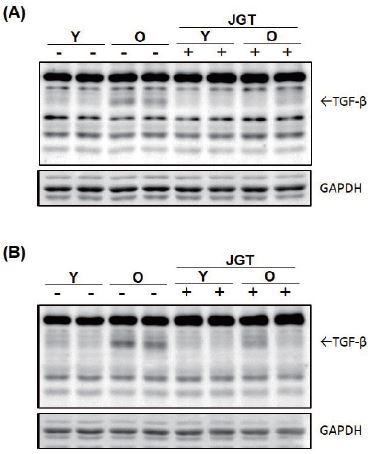
Western blot analysis of TGF-β expression in tibialis anterior (A) and gastrocnemius (B) muscles of young and old mice. GAPDH was used as the internal control. Y, young mice; O, old mice; JGT, Jaeumganghwa-tang; GAPDH, glyceraldehyde 3-phosphate dehydrogenase.

**Figure 5.**
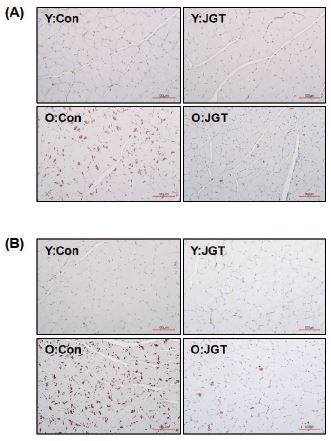
Immunohistochemical staining images of TGF-β in cross-sections of tibialis anterior (A) and gastrocnemius (B) muscles from each experimental group. Original magnification, ×200. Scale bar, 200 μm. Y:Con, young mice administered vehicle; Y:JGT, young mice administered JGT; O:Con, old mice administered vehicle; O:JGT, old mice administered JGT; JGT, Jaeumganghwa-tang.

We investigated the effect of JGT on the expression of TGF-β, one of the most potent muscle atrophy-inducing cytokines^13^. To assess whether JGT affects the local intramuscular levels of TGF-β in old mice, western blotting (Fig. 4) and immunohistochemistry were performed (Fig. 5) on the TA and GC muscles after biopsy. As expected, the intramuscular levels of TGF-β in the old mice were higher than those in the young mice. JGT treatment dramatically suppressed the expression of TGF-β in both the TA and GC muscles in the old mice. However, it had no effect on the intramuscular levels of TGF-β in the young mice groups. 

## DISCUSSION

Sarcopenia, the loss of muscle mass and function with aging, is one of the most sensitive indicators of decrepitude. It has been reported that the estimated direct healthcare expenses due to sarcopenia are 18.5 billion dollars per year in the United States^14^. Because of the global increase in the elderly population, the economic cost for sarcopenia is expected to increase exponentially.

Various interventions have been investigated for the treatment of sarcopenia. To date, exercise is the most effective strategy. It has been reported that short-term resistant exercise training maintains a modest increment in the rate of muscle protein synthesis, contributes to muscle hypertrophy, and improves muscle strength in elderly people^15^. However, when sarcopenia develops to physical impairment, it is impossible to improve the case through exercise only. Thus, in this case, alternative pharmaceutical interventions need to be considered. 

Several pharmaceutical interventions have been investigated for their ability to treat sarcopenia. For example, β-hydroxy β-methylbutyrate (HMB), a metabolite of leucine that is sold as a dietary supplement, was found to be effective in preventing muscle loss in individuals with sarcopenia^16^. Testosterone was also investigated for the treatment of sarcopenia, and seemed to have some beneficial effects on muscle strength and mass, but was not approved because of its several side effects related to sexual function^17^. No medications have been approved for the treatment of sarcopenia until now. 

Traditional herbal formulas have been used to treat various diseases for thousands of years in Korea, China, Japan, and other Asian countries^18^. Since traditional herbal formulas are generally cheaper, less toxic, and have fewer side effects than chemical drugs, they are usually applied as alternative medicines in clinical practice to treat a wide range of human diseases^19^. Recently, the potential pharmaceutical effect of traditional herbal formulas on sarcopenia has been investigated. In an experiment conducted on C2C12 muscle cell line, hachimijiogan was found to enhance skeletal muscle myoblast proliferation^20^. In addition, goshajinkigan was found to suppress sarcopenia via the insulin/IGF-1 signaling pathway. It also maintained the expression of mitochondria-related transcription factors and suppressed the expression of TNF-α in senescence-accelerated mice^21^. In addition, it further augmented resistance to exercise-induced muscle protein synthesis via the mTORC1 signaling pathway^22^. 

JGT has been prescribed for the prevention and treatment of age-related diseases for a long time. The purpose of this study was to investigate the effect of JGT on sarcopenia. Our results showed that the treatment of old mice with JGT improved muscle strength, increased skeletal muscle mass, and ameliorated muscle damage. Several factors are known to contribute to the age-related loss of muscle mass and strength. From these factors, we focused on TGF-β, which is a potent inducer of muscle atrophy^23-24^. Levels of TGF-β are elevated in various muscle diseases including sarcopenia^27^. In addition, the pathogenesis of sarcopenia involves alterations in the TGF-β signaling pathway^13^. In this study, we demonstrated that JGT treatment suppressed the intramuscular expression of TGF-β in old mice. Although further studies are still required, the beneficial effects of JGT on muscle mass and strength shown in our study suggest that it might serve as an effective treatment for sarcopenia. 
